# The Molecular Tweezer CLR01 Inhibits Antibody-Resistant Cell-to-Cell Spread of Human Cytomegalovirus

**DOI:** 10.3390/v13091685

**Published:** 2021-08-25

**Authors:** Sina Brenner, Berenike Braun, Clarissa Read, Tatjana Weil, Paul Walther, Thomas Schrader, Jan Münch, Jens von Einem

**Affiliations:** 1 Institute of Virology, Ulm University Medical Center, 89081 Ulm, Germany; sina-brenner@gmx.net (S.B.); berenike.braun@uni-ulm.de (B.B.); clarissa.read@uni-ulm.de (C.R.); 2 Central Facility for Electron Microscopy, Ulm University, 89081 Ulm, Germany; paul.walther@uni-ulm.de; 3 Institute of Molecular Virology, Ulm University Medical Center, 89081 Ulm, Germany; tatjana.weil@uni-ulm.de (T.W.); Jan.Muench@uni-ulm.de (J.M.); 4 Faculty of Chemistry, University of Duisburg-Essen, 45117 Essen, Germany; thomas.schrader@uni-due.de; 5 Core Facility Functional Peptidomics, Ulm University Medical Center, 89081 Ulm, Germany

**Keywords:** cell-to-cell spread, HCMV, tweezer, CLR01, herpesvirus, inhibition

## Abstract

Human cytomegalovirus (HCMV) uses two major ways for virus dissemination: infection by cell-free virus and direct cell-to-cell spread. Neutralizing antibodies can efficiently inhibit infection by cell-free virus but mostly fail to prevent cell-to-cell transmission. Here, we show that the ‘molecular tweezer’ CLR01, a broad-spectrum antiviral agent, is not only highly active against infection with cell-free virus but most remarkably inhibits antibody-resistant direct cell-to-cell spread of HCMV. The inhibition of cell-to-cell spread by CLR01 was not limited to HCMV but was also shown for the alphaherpesviruses herpes simplex viruses 1 and 2 (HSV-1, -2). CLR01 is a rapid acting small molecule that inhibits HCMV entry at the attachment and penetration steps. Electron microscopy of extracellular virus particles indicated damage of the viral envelope by CLR01, which likely impairs the infectivity of virus particles. The rapid inactivation of viral particles by CLR01, the viral envelope as the main target, and the inhibition of virus entry at different stages are presumably the key to inhibition of cell-free virus infection and cell-to-cell spread by CLR01. Importance: While cell-free spread enables the human cytomegalovirus (HCMV) and other herpesviruses to transmit between hosts, direct cell-to-cell spread is thought to be more relevant for in vivo dissemination within infected tissues. Cell-to-cell spread is resistant to neutralizing antibodies, thus contributing to the maintenance of virus infection and virus dissemination in the presence of an intact immune system. Therefore, it would be therapeutically interesting to target this mode of spread in order to treat severe HCMV infections and to prevent dissemination of virus within the infected host. The molecular tweezer CLR01 exhibits broad-spectrum antiviral activity against a number of enveloped viruses and efficiently blocks antibody-resistant cell-to-cell spread of HCMV, thus representing a novel class of small molecules with promising antiviral activity.

## 1. Introduction

Several enveloped viruses, including herpesviruses, have evolved a cell-to-cell mode of spread, involving direct cell–cell contacts [1]. While infection of cells with cell-free virus can efficiently be blocked by neutralizing antibodies, herpesvirus cell-to-cell spread is, with the exception of few examples [2,3], not inhibited in the presence of such antibodies [1,4,5,6,7,8].

Direct cell-to-cell spread appears to be highly relevant for herpesvirus dissemination within the infected host. Most clinical isolates of the human betaherpesvirus 5, also known as human cytomegalovirus (HCMV), grow highly cell associated in cell culture, suggesting a high importance of cell-to-cell spread for their in vivo dissemination [9,10]. Several possible mechanisms of cell-to-cell spread are proposed for various viruses, including direct transfer of mature enveloped particles between cells at tight junctions or synapses as well as the transfer of subviral particles via partial fusion of cell membranes or syncytia formation [1,11,12]. It is possible that HCMV utilizes different modes of cell-to-cell spread in different cell types, since elite human sera with outstanding HCMV neutralization capacity were partially effective against spread in endothelial cells but completely ineffective in blocking HCMV cell-to-cell spread in fibroblasts [13]. However, the exact mechanism of HCMV cell-to-cell spread is not clear. Nonetheless, it appears that HCMV cell-to-cell spread is both qualitatively and quantitatively different from cell-free infection, which supports mechanisms to evade intrinsic cellular restriction factors, IFN-induced antiviral responses, and virus neutralization by antibodies [7]. Thus, the cell-associated spread of HCMV, as well as other herpesviruses, likely represents an important mechanism to overcome immune responses. Effective antivirals that directly target the cell-associated spread would be of high therapeutic interest for the treatment of HCMV infections, since HCMV is a highly relevant opportunistic pathogen for individuals with a compromised or immature immune system, such as transplant recipients, patients with acquired immunodeficiency disease syndrome (AIDS), or connatally infected children.

Molecular tweezers are an interesting class of small, synthetic molecules that bind to amino acids, with high prevalence to lysine and arginine [14]. CLR01 was initially found to inhibit the aggregation of multiple disease-associated amyloidogenic proteins [15,16,17,18,19]. In addition, CLR01 exhibits no toxicity in mice at concentrations substantially higher than those needed for inhibition of amyloidogenic aggregates. Studies, concerning its safety and pharmacokinetics, ascribe CLR01 a high safety margin [20,21]. Later, the additional antiviral activity of the molecular tweezer CLR01 was discovered [22]. CLR01 exhibits a broad antiviral activity against a number of enveloped viruses, including the human immunodeficiency virus-1 (HIV-1), HCMV, herpes simplex virus (HSV), and hepatitis C virus. Furthermore, CLR01 has been shown to inhibit further tested enveloped viruses, such as Ebola virus, Zika virus (ZIKV), measles virus (MV), influenza A virus (IAV), and SARS-CoV-2 [23,24]. CLR01 exhibits a strong affinity to lipid head groups, which allows penetration into the membrane followed by increased surface tension and final disruption of the viral membrane [24]. Thereby, the tweezer prefers sphingomyelin binding, which is abundant in viral plasma membrane budding viruses [23,25,26,27] and could explain the selective broad antiviral activity of CLR01 against enveloped viruses.

We found that CLR01 is not only highly active in preventing the infection of cells with cell-free HCMV but also efficiently abrogates the cell-to-cell spread of HCMV and of other herpesviruses. Electron microscopy of extracellular virus particles provided evidence that, in agreement with previous findings [24], the viral envelope is a direct target of CLR01, while cell viability and virus production is unaffected. We analyzed how CLR01 affects infection by investigating different phases of viral entry and show that CLR01 impairs virus attachment and penetration. Our data not only identify the molecular tweezer CLR01 as a powerful tool against herpesviruses but also provide new insights into the mechanism of cell-to-cell spread.

## 2. Materials and Methods

### 2.1. Cell Culture and Virus Strains

Human foreskin fibroblasts (HFF) were used as described elsewhere [28,29]. HCMV strains: low-passage endotheliotropic HCMV strain TB40/E [9]; laboratory-adapted AD169 derived from pHB5-bacterial artificial chromosome (BAC); the highly fusogenic AD-UL131-repaired virus [30], reconstituted from pHB5-UL131-repaired BAC, in which a frameshift mutation in UL131 was repaired by the removal of an adenine at nucleotide position 82 by means of BAC mutagenesis (kindly provided by C. Sinzger); a dual-fluorescent HCMV strain, expressing pp150 fused C-terminally to EGFP and glycoprotein M fused at the C-terminus with mCherry (HCMV-TB40-BAC_KL7_-UL32EGFP-UL100mCherry) [31]; and a clinical HCMV isolate (first passage, 12 days in culture, derived from routine testing of patient material from the diagnostic laboratory of the Institute of Virology, Ulm University Medical Center). HSV strains of this study: an EGFP-expressing HSV-2 strain (kindly provided by P. Spear) [32] and a multi-resistant HSV-1 strain (R 10.2) (kindly provided by A. Krawczyk). Cell-free virus stocks for infection experiments were generated from supernatants of infected HFF cultivated in serum-free medium at 48 h post infection (hpi) for HSV-1 and HSV-2 and 120–168 hpi for HCMV. Supernatants were cleared from cell debris by centrifugation at 1000 rpm for 10 min and stored in aliquots at −80 °C before use.

### 2.2. Reagents and Samples

CLR01 and CLR03 were prepared as described previously [14,33]. Serum-free medium (MEM) was used in all experiments in the presence of CLR01 and CLR03. HCMV-positive urine and saliva were obtained anonymously from routine testing of patient material from the diagnostic laboratory of the Institute of Virology, Ulm University Medical Center.

### 2.3. Detection of Infected Cells

Primary monoclonal antibodies—anti-HCMV immediate early (IE) 1 (Mab63-27) [34], anti-HCMV tegument protein pp65 (Mab28-77; kindly provided by W. Britt, Univ. Birmingham, AL, USA), and anti-HSV-1 ICP0 (11060, SantaCruz Biotech, Dallas, TX, USA)—were used together with secondary goat anti-mouse antibody Alexa Fluor^®^ 488 (Invitrogen, Waltham, MA, USA) to detect infected cells in indirect immunofluorescence staining as described before [28].

### 2.4. Infection Assays

Cell-free supernatants of TB40/E (corresponding to an infection of about 50%) or patient-derived body fluids (urine or saliva), containing endogenous HCMV, were titrated with CLR01 or CLR03 (0–50 µM). After incubation for 30 min at 37 °C, virus/tweezer mixtures were added to 1.7 × 10^4^ HFF per well in 96-well flat-bottom microtiter plates for infection. In assays with urine or saliva samples, cells were washed with PBS 1 hpi and media were renewed. To determine the kinetics of CLR01, cell-free supernatants of TB40/E were incubated with 50 µM CLR01 for 0, 1, 5, 10, and 30 min. The antiviral activity of CLR01 was stopped by adding medium containing 40% fetal calf serum (FCS), resulting in a final concentration of 20% FCS. Then, HFF were incubated with these mixtures until fixation at 24 hpi and detection of the HCMV IE antigen.

### 2.5. Focus Expansion Assays

We seeded 1.7 × 10^4^ HFF per well in 96-well flat-bottom microtiter plates. Cells were infected either with cell-free HCMV (TB40/E, AD169, and AD-UL131-repaired strain) and HSV-1/2 corresponding to an infection of about 0.1–1% or by co-seeding of about 10^2^ HFF infected with a clinical HCMV isolate. Different conditions were established at either 1 day post infection or 1 day post seeding (dps) for HCMV and 1 hpi for HSV infection. Media were renewed every 48 h by washing once with PBS. Serum-free medium and an overlay medium (0.6% methylcellulose (MC), Fluka Chemie AG, Buchs, Switzerland) served as controls. Furthermore, neutralizing HCMV antibodies (Gamunex 5 mg/mL, Grifols Deutschland GmbH, Frankfurt, Germany) were used as an additional control in focus expansion assays with HCMV. Different concentrations of CLR01 were used in focus expansion assays as indicated. In the case of HSV, cells were fixed 18 hpi, and HCMV-infected cells were fixed after 6 days.

### 2.6. Cytotoxicity

For the detection of cytotoxic effects, the MTT (tetrazolium salt) assay was used according to the manufacturer’s instructions (Sigma, St. Louis, MI, USA).

### 2.7. Attachment Assay

Freshly produced cell-free supernatant of a dual-fluorescent HCMV strain was added to 2.5 × 10^5^ HFF cells in 8-well µ-slides (ibidi) for 1 h at 4 °C for attachment. Unbound virus particles were removed by washing with precooled PBS. Then, medium, neutralizing HCMV antibodies (Gamunex 5 mg/mL; Grifols Deutschland GmbH, Frankfurt, Germany), anti-HCMV gH Abs, human IgG preparations (HCMV negative, Sigma, St. Louis, MI, USA), HCMV-positive elite human serum [35], and different concentrations of CLR01 as indicated were added for 1 h at 4 °C before cells were shifted to 37 °C. Infection was determined the next day by staining for HCMV IE antigen. For quantification of attached HCMV particles after CLR01 treatment, cell-free supernatants of the dual-fluorescent HCMV strain were pretreated with CLR01 for 30 min at 37 °C. Then, mixtures were cooled to 4 °C and added to HFF for 1 h at 4 °C. Unbound virus particles were removed by washing with precooled PBS, and, subsequently, cells were fixed with 80% acetone for 10 min at room temperature. Cell nuclei were visualized with 4′6-diamidino-2-phenylindole (DAPI, Roche, Basel, Switzerland). Attached HCMV particles per cell were quantified via the mCherry signal of the labeled envelope protein M.

### 2.8. RT-qPCR

The release of HCMV genomes during infection in the presence of CLR01 was controlled using real-time quantitative polymerase chain reaction (RT-qPCR). We infected 1.7 × 10^4^ HFF per well in 96-well flat-bottom microtiter plates with HCMV strain TB40/E (corresponding to an infection of about 50%) for 1 h at 37 °C. Infected cells were cultured from 1 dpi in either medium or medium containing CLR01 (50 µM). Supernatants collected at 1, 4, and 6 dpi were digested with proteinase K (Roche) for 1 h at 56 °C followed by incubation for 5 min at 95 °C. Then, samples were used for RT-qPCR as described previously [36,37].

### 2.9. Electron Microscopy

To visualize the effect of CLR01 on virion structure, transmission electron microscopy (TEM) of extracellular HCMV virions was performed using a high-pressure freezing and freeze-substitution protocol as previously described [38]. Briefly, fibroblasts were grown on sapphire discs (Wohlwend GmbH) and infected with an MOI of 0.01 plaque-forming units (pfu)/mL. On day 5 post infection, the cells were immobilized by high-pressure freezing (HPF Compact 01, Wohlwend GmbH) and processed for TEM by freeze substitution (0.1% uranyl acetate, 0.2% osmium tetroxide, and 5% water in acetone over 17 h), embedding in Epon, and ultrathin sectioning to a thickness of 70 nm (Reichert Ultracut S equipped with a diamond knife from Diatome). Images were taken at 120 kV acceleration voltage on a JEM-1400 TEM (Jeol, Tokyo, Japan).

### 2.10. Quantifications and Statistical Analysis

For quantification of HCMV infection, the “Image-based Tool for Counting Nuclei” from ImageJ (https://imagej.nih.gov/ij/plugins/itcn.html access on 18 February 2021) was utilized. The following parameters were adjusted: width: 30 pixels; minimum distance: 18 pixels; and threshold: 0.1. Infection was quantified from 2 × 2 mosaic images, obtained with the 10× objective of Axio-Observer.Z1 fluorescence microscope (Zeiss, Jena, Germany). For quantification of attached HCMV particles per cell, 3 × 3 mosaic images were obtained with the 63× objective. Viral particles per cell were determined for at least 100 cells per condition from two individual experiments. HSV infection (infected area) in focus expansion assays was quantified from 2 × 2 mosaic images obtained with the 10× objective and the Zen2.3 analysis software (Zeiss, Jena, Germany). Half-maximal inhibitory concentrations were determined graphically using Microsoft Excel. The software GraphPad Prism 5.01 was utilized for statistical analysis. Data significance levels were calculated using one-way analysis of variance (ANOVA, San Francisco, CA, USA) (non-parametric), followed by Bonferroni’s multiple comparison test. *p* values of <0.01 were considered significant.

## 3. Results

### 3.1. The Molecular Tweezer CLR01 Inhibits HCMV Infection

**Figure 1:**
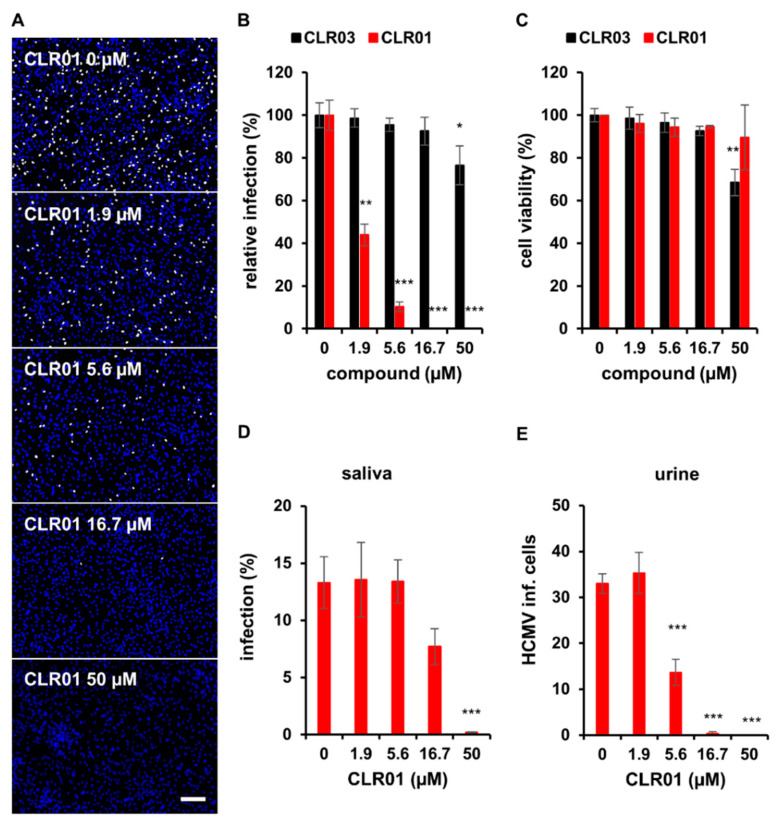
CLR01 inhibits HCMV infection. (**A**,**B**) Cell-free virus particles of HCMV (strain TB40/E, corresponding to an infection of about 50%) were incubated with indicated concentrations of CLR01 or CLR03 for 30 min at 37 °C prior to infection of human fibroblasts with these mixtures. Infection was determined by indirect immunofluorescence of HCMV IE antigen 24 hpi. (**A**) Representative images of infected cells: blue, DAPI-positive cells; white, HCMV IE-positive cells. Scale bar is 200 µm. (**B**) Means ± SDs of relative infection are from the combined results of three individual infection experiments, each performed in triplicate. (**C**) Cytotoxicity of indicated concentrations of CLR01 and CLR03 on human fibroblasts was controlled after 24 h incubation using the MTT assay. Means ± SDs from two individual experiments, each performed in triplicate. Controls (0 µM) were set to 100% and samples normalized accordingly. (**D**,**E**) HCMV-positive urine and saliva (undiluted) were incubated with indicated concentrations of CLR01 for 30 min at 37 °C prior to infection of human fibroblasts with these mixtures for 1 h at 37 °C. Cells were stained for HCMV IE antigen by indirect immunofluorescence at 24 hpi. (**D**) Means ± SDs of infection (%) and (**E**) number of HCMV-positive cells per well from triplicate infections. *, *p* < 0.01; **, *p* < 0.001; ***, *p* < 0.0001.

The molecular tweezer CLR01 has been shown to exhibit broad antiviral activity against several enveloped viruses, including human cytomegalovirus (HCMV) [22,23]. Here, we analyzed the antiviral activity of CLR01 against HCMV in more detail and compared it to CLR03, a control that lacks the tweezers’ side arms [16] and the antiviral activity [22,23]. In vitro-derived cell-free virus (HCMV strain TB40/E) was incubated with different concentrations of tweezers for 30 min at 37 °C prior to inoculation of monolayer cultures of human fibroblasts with the virus–tweezer mixtures. After an incubation of 24 h, HCMV immediate early (IE) antigen-positive cells were detected as a marker for viral entry and the initiation of replication. CLR01 inhibited HCMV infection of human fibroblasts dose dependently (IC_50_ 1.70 ± 0.76 µM) and was able to block infection completely at concentrations >16.7 µM (Figure 1A,B). Importantly, cell viability was not significantly affected by CLR01 even at the highest concentration tested (Figure 1C). As previously shown, CLR01 directly targeted virus particles and did not affect the susceptibility of cells to infection with HCMV when cells were pretreated with CLR01 prior to infection (Supplementary Figure S1A) [22]. By contrast, CLR03 did not inhibit infection, demonstrating and verifying the specific antiviral effect of CLR01 (Figure 1B). Unlike CLR01, CLR03 exhibited significant cytotoxicity on human fibroblasts at concentrations above 16.7 µM and, therefore, was not a suitable control in assays that required higher concentrations (Figure 1C).

Next, we addressed the question of whether CLR01 is active against endogenous virus in body fluids of HCMV-infected patients. Therefore, HCMV-positive urine from an infected newborn and HCMV-positive saliva from an infected adult were incubated with different concentrations of CLR01, and infection assays were performed as described above. The infection of human fibroblasts from HCMV-positive saliva (Figure 1D) as well as urine (Figure 1E) was inhibited dose dependently by CLR01. This shows that CLR01 is active against clinical HCMV strains in patient-derived material and can protect cells from virus infection.

### 3.2. CLR01 Has a Fast-Acting Mode of Action That Prevents Entry at Various Steps

**Figure 2:**
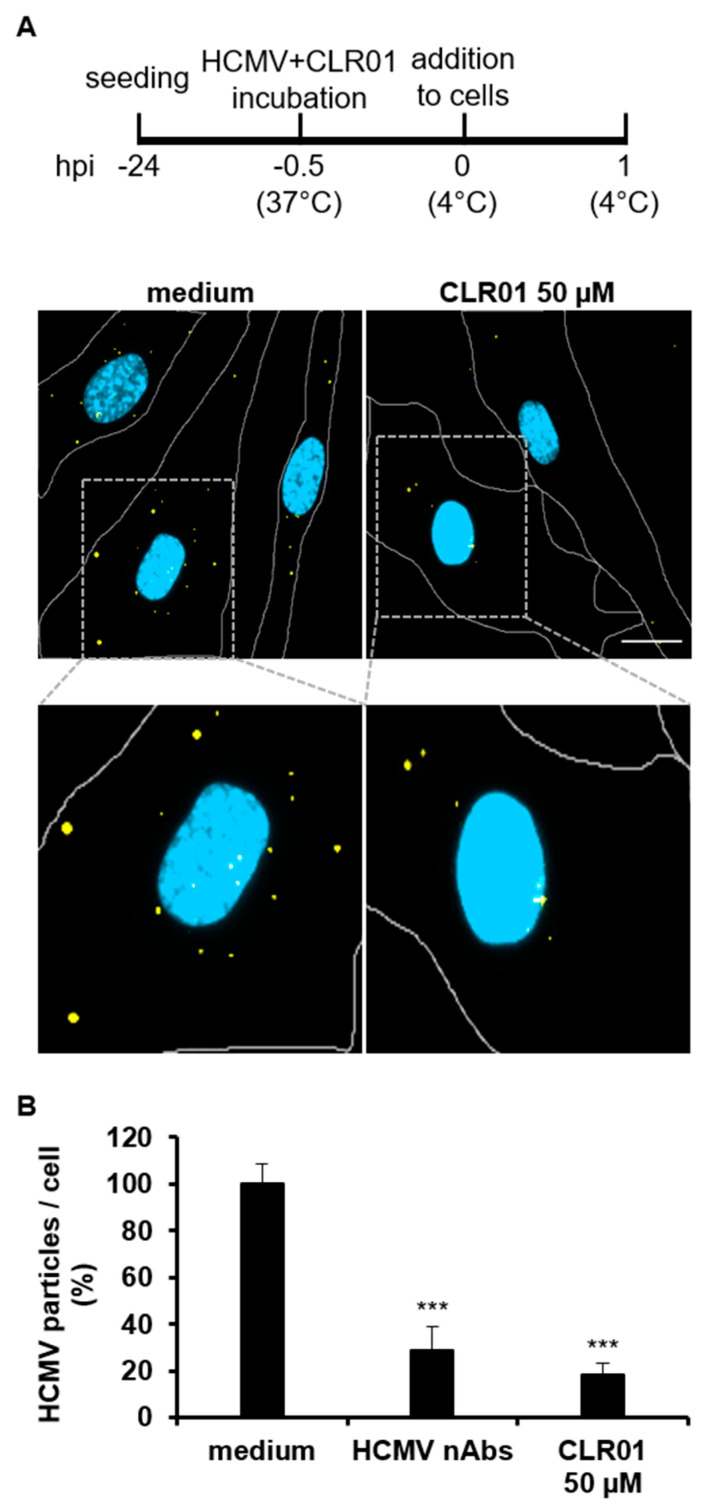
CLR01 reduces the attachment capacity of HCMV particles. (**A**) Cell-free virus particles of a fluorescent-tagged HCMV were incubated with 50 µM CLR01 and medium for 30 min at 37 °C. Attachment of virus particles to human fibroblasts was allowed for 1 h at 4 °C. Blue, DAPI-stained cell nuclei; yellow, cell-bound HCMV particles; and white, cell borders. Scale bar is 5 µm. (**B**) Quantification from A with neutralizing antibodies (nAbs, 5 mg/mL Gamunex) as an additional control. Number of attached HCMV particles per cell was determined for at least 100 cells for each condition from two individual experiments. Means ± SDs of relative numbers of attached HCMV particles per cell. Controls (medium) were set to 100% and samples normalized accordingly. ***, *p* < 0.0001.

We and others have shown that CLR01 directly targets viral particles (Supplementary Figure S1A) [22,23] and particularly the viral membrane [24]. To better understand the consequences of CLR01 binding to the HCMV envelope, we investigated which entry step of HCMV is inhibited by CLR01. HCMV entry can be separated into two distinct phases: attachment and penetration. Attachment was examined using cell-free fluorescence-tagged HCMV particles, which were incubated with either 50 µM CLR01, neutralizing antibodies (nAbs), or medium for 30 min at 37 °C before addition to human fibroblasts. Cells were incubated with the differently treated virus particles for 1 h at 4 °C, which allows adsorption of the particles to the cell surface but suppresses the subsequent steps of virus entry [39]. The attachment of virus particles to human fibroblasts was quantified by fluorescence microscopy after unattached virus particles were removed (Figure 2A). CLR01 reduced the number of attached virus particles by about 70% as compared to the medium control (Figure 2B). A similar reduction in the attachment capacity of virus particles was observed in the presence of nAbs, which were used at a concentration that completely blocked cell-free infection in infection assays (Supplementary Figure S1B). This indicated that CLR01 impairs the ability of HCMV particles to attach to the cell surface.

**Figure 3:**
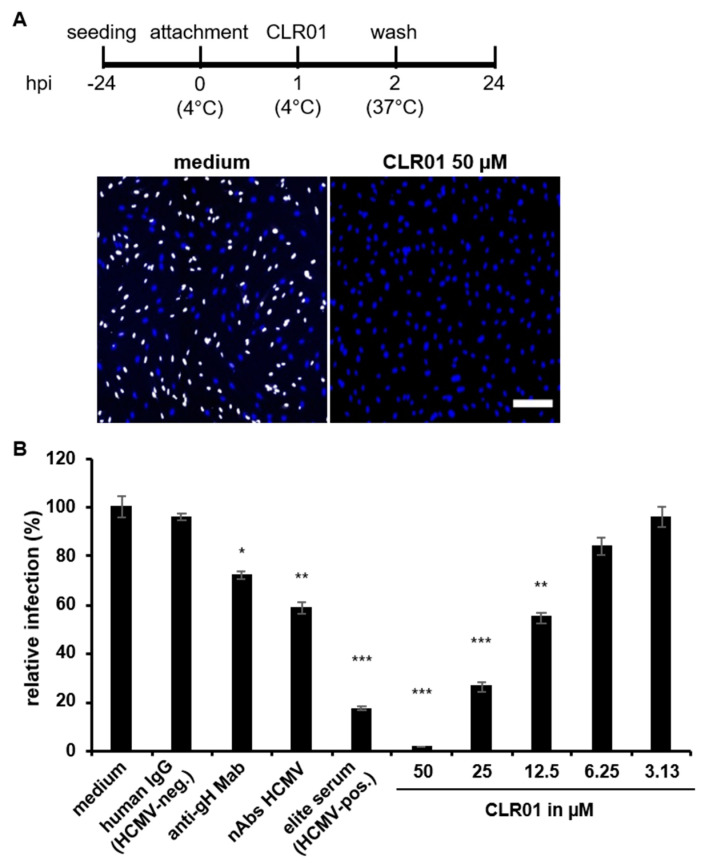
CLR01 blocks viral infection post attachment. (**A**) Cell-free virus particles of HCMV (strain TB40/E) were attached to human fibroblasts for 1 h at 4 °C prior to addition of either 50 µM CLR01 or medium for another hour at 4 °C. Infection was determined at 24 hpi by detection of HCMV IE antigen after the shift to 37 °C. Blue, DAPI-positive cells; white, HCMV-positive cells. Scale bar is 200 µm. (**B**) Quantification from A with additional controls (neutralizing Abs (nAbs, 5 mg/mL Gamunex), anti-gH Abs (10 µg/mL), human IgG (HCMV negative, 10 µg/mL), elite human serum (HCMV positive, 1:10)), and indicated concentrations of CLR01. Means ± SDs of relative infection are from the combined results of two individual experiments, each performed in triplicate. Controls (medium) were set to 100% and samples normalized accordingly. *, *p* < 0.01; **, *p* < 0.001; ***, *p* < 0.0001.

Next, we analyzed whether CLR01 is also able to inhibit virus entry post attachment. Therefore, HCMV particles were first attached to human fibroblasts at 4 °C and subsequently incubated with CLR01, medium, or nAbs. The cells were then transferred to 37 °C to allow virus penetration and hence infection. Infection was measured after 24 h by detection of the HCMV IE antigen. The addition of CLR01 to already attached virus particles inhibited infection in a dose-dependent manner, demonstrating that CLR01 is capable of inhibiting the post attachment steps of HCMV entry. In contrast, Gamunex, a commercially available formulation of human immunoglobulins with nAbs against HCMV, reduced infection by about 30–40% compared with the medium control and compared with human immunoglobulins (IgG, HCMV negative), the latter used as a control for non-HCMV-specific effects of antibodies on virus penetration (Figure 3A,B). Because of the poor efficacy of Gamunex in inhibiting HCMV penetration, other anti-HCMV antibodies were tested for their ability to inhibit virus penetration. These included a monoclonal antibody against HCMV glycoprotein H (gH) as an antibody with a defined target and a recently described human serum from an elite neutralizer that exhibits broad and potent neutralization against multiple HCMV strains, particularly inhibiting penetration [35]. All anti-HCMV antibodies were used at a concentration at which cell-free infection was completely blocked (Supplementary Figure S1C,D). As shown in Figure 3B, anti-gH Abs were able to reduce infection by 30%, which is comparable to the mixture of anti-HCMV antibodies in Gamunex. The human serum from the elite neutralizer, however, reduced infection by 80%. Taken together, the antiviral activity of CLR01 affects not only virus attachment but also post attachment entry steps of HCMV. CLR01 was able to effectively inhibit infection by already attached viral particles in contrast to antibodies that could only partially inhibit infection.

**Figure 4:**
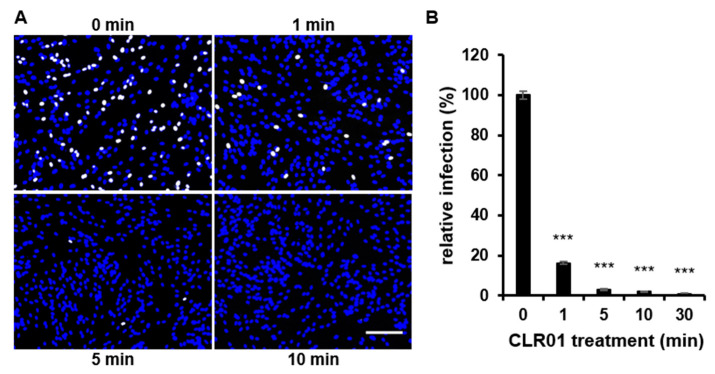
CLR01 rapidly inactivates HCMV particles. Cell-free virus particles of HCMV (strain TB40/E) were incubated with 50 µM CLR01 for indicated times at 37 °C prior to inactivation of CLR01 by adding medium containing fetal calf serum (FCS; final concentration 20%) and infection of human fibroblasts with these mixtures. Infection was determined after detection of HCMV IE antigen by indirect immunofluorescence at 24 hpi. (**A**) Blue, DAPI-positive cells; white, HCMV-positive cells. Scale bar is 200 µm. (**B**) Means ± SDs of relative infection are from the combined results of two individual experiments, each performed in triplicate. Control (0 min) was set to 100% and samples normalized accordingly. ***, *p* < 0.0001.

We next sought to investigate the kinetics of the antiviral effect of CLR01 on virus particles. For this, the antiviral activity of CLR01 was inactivated by the addition of serum at different times after incubation of cell-free virus particles with CLR01 [23]. The remaining infectivity was determined on human fibroblasts in infection assays. Strikingly, the infectivity of cell-free virus particles was reduced by approximately 80% after only 1 min of CLR01 and continued to decrease with longer CLR01 treatment (Figure 4). Overall, these data demonstrate that CLR01 is a fast-acting antiviral agent that inhibits viral entry in both the attachment and penetration steps.

### 3.3. CLR01 Inhibits Direct Cell-to-Cell Spread of HCMV

**Figure 5:**
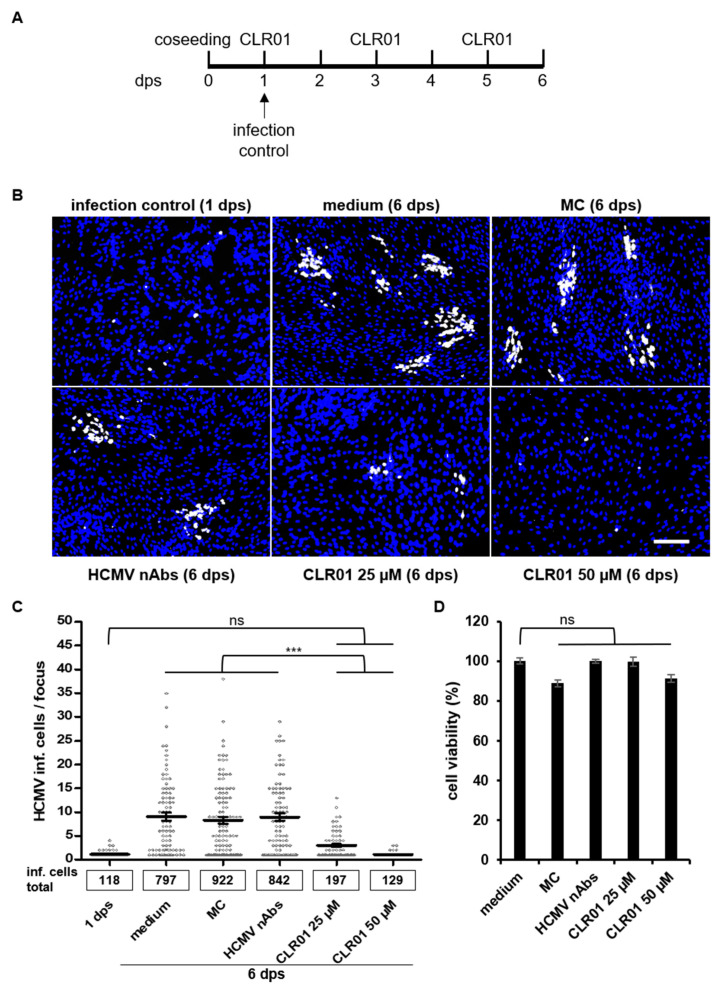
CLR01 inhibits direct cell-to-cell spread of a clinical HCMV isolate. (**A**) Human fibroblasts were seeded together with cells infected with a clinical HCMV isolate. Different conditions (medium, methylcellulose (MC) overlay, HCMV-neutralizing antibodies (nAbs; 5 mg/mL Gamunex), and indicated concentrations of CLR01) were applied 1 day post seeding (dps) and renewed every 48 h. Focal growth was determined by indirect immunofluorescence of HCMV IE antigen at 6 dps. Initial infection was controlled by detection of HCMV-positive cells at 1 dps. (**B**) Blue, DAPI-positive cells; white, HCMV-positive cells. Scale bar is 200 µm. (**C**) Means ± SEMs. Each data point represents the number of HCMV-infected cells per focus quantified from images from B. (**D**) Cell viability of human fibroblasts cultivated under indicated conditions for 6 days at 37 °C was controlled using the MTT assay. Means ± SDs are from the combined results of two individual experiments, each performed in triplicate. Controls (medium) were set to 100% and samples normalized accordingly. ***, *p* < 0.0001.

Direct cell-to-cell spread is important for HCMV dissemination in vivo. This is also demonstrated by the strictly cell-associated growth of clinical isolates in cell culture after their primary isolation from patient material [9]. Currently, there is no treatment that can directly inhibit the cell-to-cell spread of HCMV. Neutralizing antibodies produced in response to infection can efficiently block the infection of cells via cell-free viruses but mostly fail to prevent direct cell-to-cell spread [6,13]. The molecular tweezer CLR01 seems to have superior properties in preventing penetration of attached virus particles in comparison to nAbs and also shows a rapid antiviral effect on viral particles. Therefore, we investigated whether CLR01 is able to affect the cell-to-cell spread of HCMV. Focus expansion assays were performed using human fibroblasts that were seeded together with cells infected with a clinical HCMV isolate that spreads by cell-to-cell transmission only. Different concentrations of CLR01, nAbs, methylcellulose (MC) overlay medium (which prevents virus spread by cell-free virus [40]) or medium only were added 24 h post seeding (hps). Cell-to-cell spread was examined after six days by detection of HCMV IE antigen-positive cells (Figure 5A,B). The initial infection was controlled at 24 hps and, as expected, showed only single infected cells (Figure 5B, infection control). Further cultivation of the clinical isolate resulted in foci of varying sizes with an average of ten IE-positive cells at 6 days post seeding (dps) when grown in medium only (Figure 5B,C). Foci in the presence of MC and nAbs were of similar size than those of medium alone, which is consistent with the highly cell-associated growth of the clinical HCMV isolate. It also showed the inability of nAbs to inhibit HCMV cell-to-cell spread. In contrast, cultivation in the presence of 25 µM CLR01 resulted in smaller foci with a mean size of three IE-positive cells per focus, indicating that cell-to-cell spread is impaired. Most strikingly, HCMV cell-to-cell spread was completely inhibited in the presence of 50 µM CLR01, because only single IE-positive cells were found after 6 days. The number of infected cells at 6 dps with 50 µM CLR01 was similar to the initial number of infected cells at 24 hps (Figure 5C). Of note, the prolonged incubation of cells for five days with 50 µM CLR01 did not affect cell viability (Figure 5D). Hence, CLR01 prevents cell-to-cell spread of a clinical HCMV isolate.

**Figure 6:**
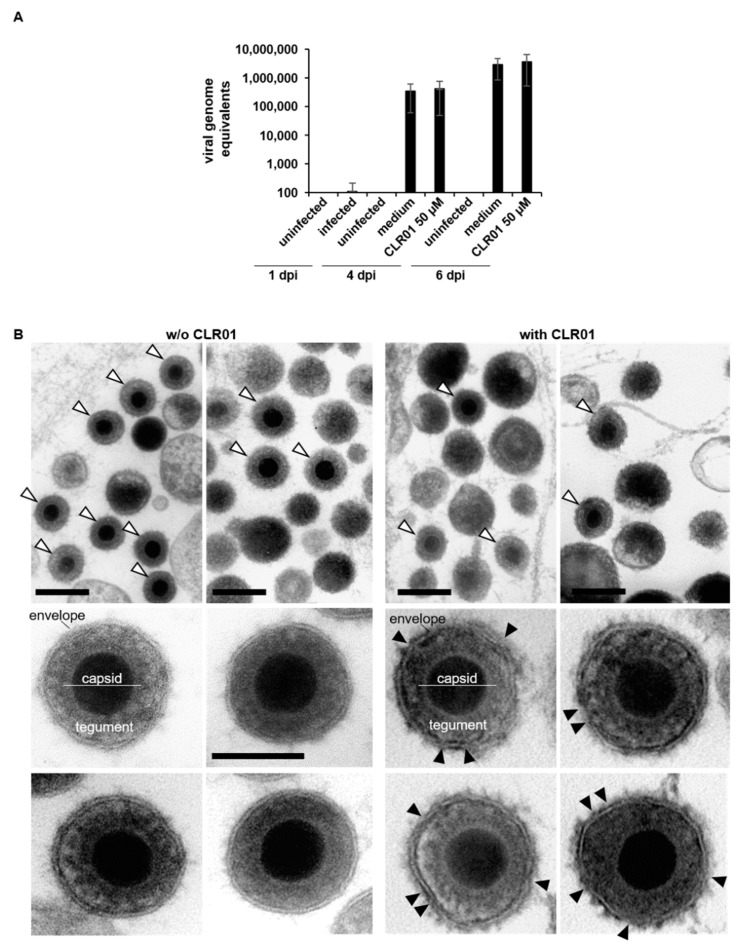
CLR01 does not impair HCMV release during infection but alters virion structure. (**A**) Human fibroblasts were infected with cell-free TB40/E (corresponding to an infection of about 50%) for 1 h and cultured from 1 dpi in either medium or medium containing CLR01. Supernatants were harvested 1, 4, and 6 dpi followed by proteinase K digestion and subsequently used for RT-qPCR. (**B**) Transmission electron microscopy of extracellular HCMV virions after 6 days of cultivation on human fibroblasts without (left) and with 50 µM CLR01 (right) according to the focus expansion protocol (Supplementary Figure S2). First row: overviews of virions (white arrowheads) in the extracellular space. Scale bar 200 nm. Lower rows: higher magnifications of extracellular virions. Note the circular and regular appearance of virions without CLR01 compared to the altered appearance of virions when treated with CLR01, including the extra layer of electron dense material lining their envelope. Black arrowheads: discontinuities and indentations of the viral envelope. Scale bar: 100 nm.

Although CLR01 neither affects cell viability nor their susceptibility to HCMV infection at concentrations sufficient to block cell-to-cell spread, we sought to exclude the possibility that CLR01 affects virus production or release during infection. Therefore, human fibroblasts were infected with HCMV strain TB40/E that, unlike the clinical HCMV isolate, releases infectivity into the supernatant. Released virus particles in supernatants of infected cells were quantified by real-time quantitative polymerase chain reaction (RT-qPCR) at 1, 4, and 6 dpi. We measured similar amounts of HCMV genomes in supernatants of medium- and CLR01-treated cells (Figure 6A), indicating that the presence of CLR01 does not impair virus production and release.

To learn more about the mechanism of the anti-HCMV activity of CLR01, we next tried to visualize virus particles by electron microscopy (EM). It has been shown previously that CLR01 affects the integrity of the viral membrane and disrupts HIV-1 and Zika virus particles [22,23,24]. To investigate the effect of CLR01 on HCMV under our experimental conditions, TB40/E-infected cells were cultivated under the conditions of a focus expansion assay (Supplementary Figure S2) and subsequently analyzed by EM. Numerous virus particles were found in the extracellular space of cells treated with CLR01 and medium, which confirms our RT-qPCR data and shows that CLR01 does not affect the production and release of virus particles (Figure 6B). At higher magnification, the envelope of extracellular HCMV particles in CLR01-treated samples appeared to be decorated with electron dense material, which was not observed for extracellular virus particles of medium-treated samples. Furthermore, the lipid bilayer of the viral envelope seemed to be partially disrupted in the presence of CLR01.

**Figure 7:**
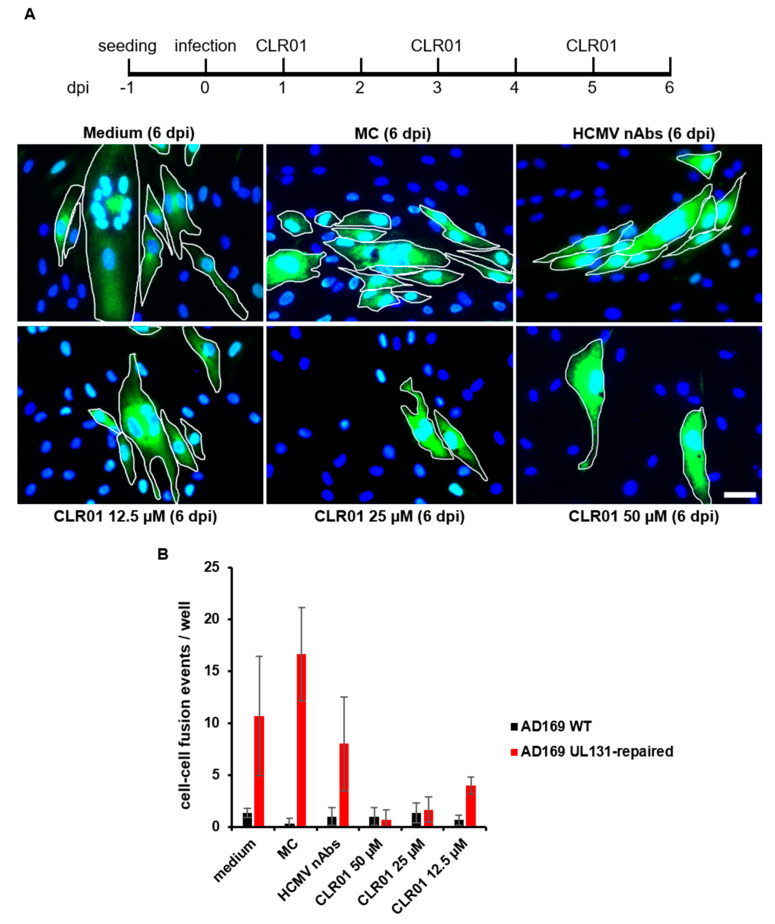
CLR01 inhibits cell–cell fusion of cells infected with AD169 UL131-repaired HCMV strain. (**A**) Human fibroblasts were infected with AD169 UL131-repaired HCMV strain (corresponding to an infection of about 0.1–1%). Different conditions (medium, methylcellulose (MC) overlay, HCMV-neutralizing antibodies (nAbs; 5 mg/mL Gamunex), and indicated concentrations of CLR01) were applied 1 dpi and renewed every 48 h. Focal growth and the formation of cell–cell fusion were determined by indirect immunofluorescence of HCMV tegument protein pp65 at 6 dpi. Blue, DAPI-positive cells; green, HCMV-positive cells; white, cell borders. Scale bar is 50 µm. (**B**) Focus expansion assays were performed with AD169 and AD169 UL131-repaired strain as described in A. Cell–cell fusion events were quantified from triplicate infection. A cell–cell fusion event was defined as at least two cell nuclei within one cell, whereby the different numbers of cell nuclei within cell–cell fusion events was not considered.

The cell-to-cell spread of HCMV is a complex process that is not completely understood. Several possible modes are proposed and discussed [1,11]. Besides the release of enveloped and infectious virus particles at cell junctions, cell–cell fusion is also a conceivable mechanism of HCMV cell-to-cell spread. To test whether CLR01 can also block spread by cell–cell fusion, we used the AD-UL131-repaired HCMV strain in focus expansion assays. The viral growth of the AD-UL131-repaired strain is characterized by enhanced membrane fusion and syncytium formation in human fibroblasts [30]. Indeed, several multinucleated cells, indicative for virus-induced syncytium formation, were detected for the AD-UL131-repaired strain after cultivation for 6 days (Figure 7). Neither MC nor nAbs blocked syncytia formation. In contrast, the cultivation of the AD-UL131-repaired strain in the presence of 50 µM of CLR01 resulted in only single infected cells and apparently blocked cell–cell fusion, as multinucleated cells were not detected (Figure 7B). Collectively, these data show that the molecular tweezer CLR01 is able to block antibody-resistant cell-to-cell spread of HCMV.

### 3.4. Inhibition of Herpesvirus Cell-to-Cell Spread by CLR01 Is Not Restricted to HCMV

**Figure 8:**
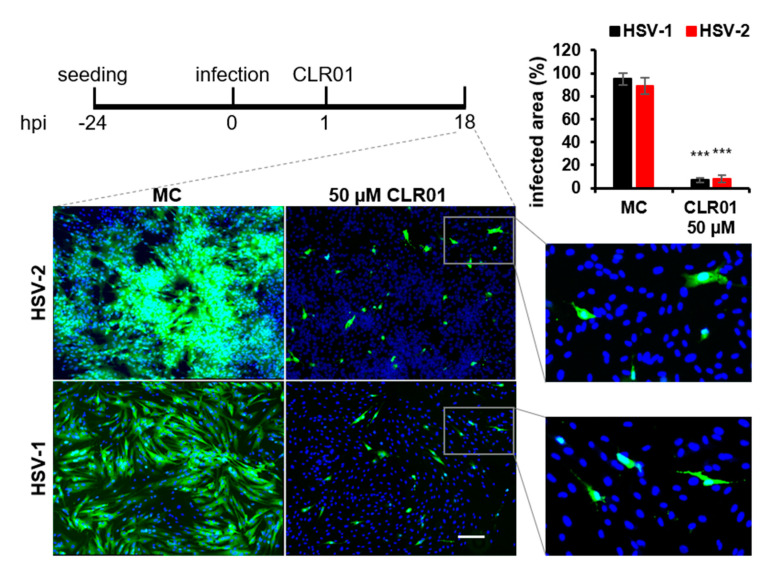
CLR01 inhibits cell-to-cell spread of HSV-1 and HSV-2. Human fibroblasts were infected with indicated viruses (corresponding to an infection of about 0.1–1%). Methylcellulose (MC) overlay or 50 µM CLR01 were added at 1 hpi, and the virus spread was examined at 18 hpi. Virus dissemination was determined from the GFP signal of HSV-2-infected cells and from indirect immunofluorescence staining of ICP0 of HSV-1-infected cells. Blue, DAPI-positive cells; green, HSV-positive cells. Scale bar is 200 µm. Means ± SDs of infected area (%) quantified from at least three randomly taken images for each condition. ***, *p* < 0.0001.

Direct cell-to-cell transmission is a mechanism of virus dissemination that is also used by other herpesviruses. Therefore, we tested whether CLR01 can also block cell-to-cell spread of the alphaherpesviruses HSV-1 and -2. These viruses were chosen because CLR01 was shown to inhibit the infection of cells by cell-free HSV-2 [22] and both viruses are known to spread in a cell-associated manner in the presence of nAbs [41]. HSV-1 and HSV-2 have a short replication cycle releasing viral progeny after 6 hpi [42]. Therefore, cell-to-cell spread of HSV-1 and -2 was determined in a focus expansion assay by infection of human fibroblasts for 1 h at 37 °C followed by an incubation with either 50 µM CLR01 or MC for an additional 18 h. The incubation of cells with 50 µM CLR01 resulted in only single infected cells, whereas large foci were formed in the presence of MC (Figure 8). Hence, CLR01 effectively prevented direct cell-to-cell spread of the alphaherpesviruses HSV-1 and -2 in human fibroblasts.

**Figure 9:**
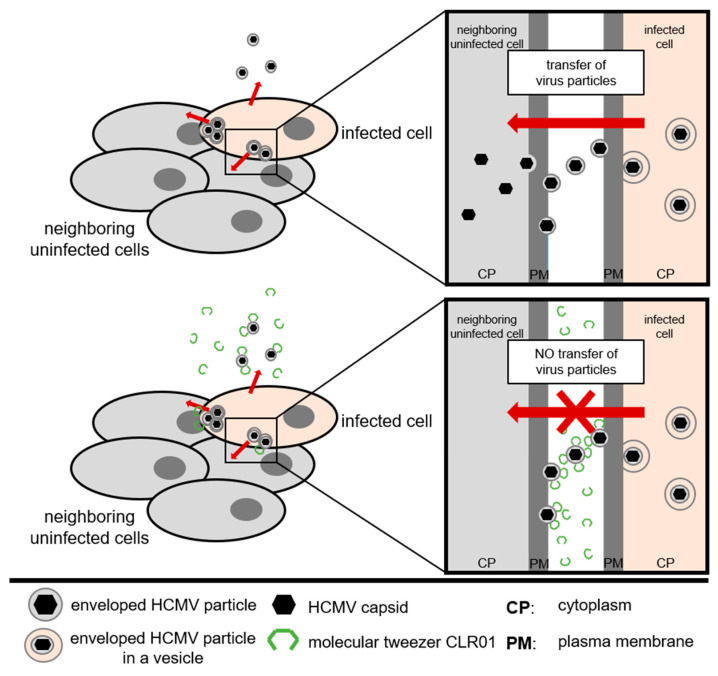
Model of HCMV cell-to-cell spread in human fibroblasts. Upper panel: Unrestricted transfer of enveloped HCMV particles between cell–cell contacts. Lower panel: CLR01 blocks transfer of enveloped HCMV particles between cell–cell contacts.

Taken together, these data suggest that the rapid and efficient inactivation of enveloped herpesvirus particles by CLR01 at various steps of viral entry may also inhibit the cell-to-cell spread of HCMV and other herpesviruses (Figure 9).

## 4. Discussion

### 4.1. CLR01 Inhibits HCMV Entry

Our results extend previous findings demonstrating that CLR01 acts as an effective antiviral agent against herpesvirus infections by directly targeting the envelope of cell-free virus particles. We could show that the disruption of the viral envelope by CLR01 leads to a significant reduction of both, attachment and penetration, and can thus efficiently inhibit viral entry. CLR01 was active in saliva and urine and prevented infection of susceptible cells by endogenous HCMV. This highlights CLR01 as a promising candidate for the treatment of herpesvirus infections, also due to its high safety and low toxicity in mouse model studies [21]. Our data support the previously proposed antiviral mode of action by which CLR01 selectively binds to and disrupts viral particles [22,23]. Cryo-TEM of HCMV particles has also shown that CLR01 compromises the integrity of the viral envelope and causes partial ruptures in the viral membrane [24]. Here, electron micrographs of released HCMV particles under focus expansion assay conditions also appeared to have a disrupted viral envelope and were additionally surrounded by electron dense material, reminiscent of the Cryo-TEM data. Hence, the viral envelope seems to be the main target of CLR01. Since the viral envelope originates from the infected cell, interaction of CLR01 with cellular membranes cannot be ruled out. However, cell viability was not affected by CLR01 in our experiments, even after several days of cultivation, and, as mentioned earlier, no cytotoxicity to CLR01 was observed in studies on mouse models at concentrations well above those used in this study [20,21]. It is conceivable that cells are able to repair potential membrane damage caused by CLR01, whereas virions are unable to do so. In conclusion, our data underline previous work on the importance of the viral envelope, and cholesterol in particular [43], for herpesvirus entry and further demonstrate that the viral envelope is a promising antiviral target and that compounds directed against it, such as CLR01, are effective against many enveloped viruses.

### 4.2. CLR01 Potently Blocks Antibody-Resistant Cell-to-Cell Spread of HCMV

The most remarkable result of this study was that CLR01 was able to effectively block the cell-to-cell spread of HCMV and HSV-1 and -2 in cell cultures. In addition to its antiviral potential, the inhibition of antibody-resistant cell-to-cell spread by CLR01 in the context of the tweezer’s mode of action provides valuable insights into the mechanism of this mode of virus dissemination in HCMV and also in other herpesvirus infections. The mechanism of the direct cell-to-cell spread of HCMV is not fully understood [1,11]. It has been established that cell–cell contacts are required [7] and that cytoplasmic material can be transferred from infected cells, most likely through cell–cell fusion [44]. In addition, virus lacking an essential tegument protein (pUL99) required for secondary envelopment and release of infectious cell-free virus particles can still form foci of infected cells in fibroblasts [45]. Furthermore, the transmission of viral particles can proceed differently in different cell types [46]. This indicates that there is not only one mechanism of cell–cell transmission and that further investigations are needed to fully elucidate them. One possible mechanism of cell-to-cell spread is via the release of infectious enveloped virus particles at cell junctions and infection of the adjacent cell. Induction of cell–cell fusion as, e.g., syncytia formation, represents another mode of cell-to-cell spread. Recently, a new form of cell-to-cell contacts called tunneling nanotubes (TNTs) has been described, which is used by alphaherpesviruses for cell-to-cell spread even in the presence of nAbs [12]. The transfer of virus via TNTs does not require the release of enveloped virions and could be used by the virus to pass to neighboring cells without the exposure of virus particles to the extracellular space. However, it is not entirely clear to what extent cell–cell fusion events and the transfer of premature virions are relevant for the spread of herpesviruses in vivo. This also means that the necessity of an envelope for herpesvirus cell-to-cell spread has not been sufficiently clarified. However, it is known that viral glycoproteins have an important role in herpesviral cell–cell spread. It has been shown that the alphaherpesvirus envelope glycoproteins gB, gD, gE/gI, and gH/gL are required for cell-to-cell spread [47,48]. In addition, HCMV gB has been shown to be essential for virus entry and spread [49]. Furthermore, involvement of glycoprotein complexes, e.g., the pentameric and trimeric complexes, in the HCMV cell–cell spread has been shown [7,50]. Nevertheless, it is not known whether these glycoproteins must be part of the viral envelope or whether their expression on the cell surface is sufficient [51]. Our results suggest that the cell-to-cell spread of HCMV in fibroblasts mainly requires enveloped virus particles on the basis of CLR01’s mode of action and its total prevention of cell-to-cell spread. Admittedly, this reflects the experimental conditions of this investigation and cannot be transferred directly to in vivo circumstances.

CLR01 also inhibited cell–cell fusion of the AD169 UL131-repaired variant. The fusogenic phenotype of the AD169 UL131-repaired strain is likely due to higher fusion activity of the gB(275Y) variant in AD169 in cooperation with the pentameric complex gH/gL/UL128-131 [52]. The release of viral particles, particularly at cell–cell contacts, could lead to the fusion of cell membranes and thus syncytium formation due to higher fusion activity of gB, as in the case of AD169. Such a conceivable viral particle-mediated mechanism could explain the inhibition of cell fusion by CLR01. In this context, it would also be interesting to test whether CLR01 can also inhibit syncytium formation of recently described clinical isolates of HCMV [53].

HCMV cell-to-cell spread in the presence of nAbs can have several reasons. One possibility is that due to the site of spread, e.g., at secluded cell–cell contacts, these places are difficult to access for antibodies or they are sterically hindered [1]. CLR01, compared to antibodies, is a very small molecule and, therefore, may be able to reach these areas in sufficient quantity. However, there are also examples of inhibition of cell-to-cell spread by antibodies. These often appear to be specific antibodies, e.g., broadly neutralizing antibodies that block cell-to-cell spread of HIV [54]. Furthermore, an engineered glycoprotein B (gB)-specific monoclonal antibody and some antibodies against gD are able to block cell-to-cell spread of HSV-1 and -2 [2,3,29], which argues against steric hindrance. When enveloped virus particles are released into the narrow gap between two adjacent cells, it is possible that these particles never loose cell contact and immediately attach to the neighboring cell. Notably, nAbs were not able to block the infection of cells with already attached viral particles, whereas CLR01 was very effective in infection inhibition of the attached virus. Considering our data from the post attachment assays, it is conceivable that most antibodies cannot block cell-to-cell spread because the virions may already be attached to the neighboring cell and thus their subsequent entry steps cannot be prevented. Only a selected and highly active human serum derived from a broad screening approach to identify sera with outstanding HCMV neutralization capacity [35] was able to substantially reduce infection in our post attachment assays. This highlights the importance of post attachment steps during virus entry, which may also be a promising target in cell-to-cell spread. However, the ability of antibodies to inhibit post attachment steps in particular does not seem to be sufficient in inhibiting the cell-to-cell spread of HCMV in fibroblasts, because the elite neutralizing human serum and also other sera from the screening approach are not able to block the cell-to-cell spread of HCMV in fibroblasts [13]. Interestingly, these sera were partially effective against HCMV cell-to-cell spread in endothelial cells, suggesting that HCMV may use different pathways of cell-to-cell spread in different cell types. Nevertheless, HCMV cell-to-cell spread in fibroblasts, which are important target cells in the human host, was blocked by CLR01 and not by nAbs. Hence, the molecular tweezer CLR01 seems to have advantageous properties over nAbs in preventing the cell-to-cell spread of HCMV and also of other herpesviruses. In addition to the size of the tweezer, its mode of action certainly plays a crucial role. The interaction of CLR01 with virions leads to disruption of the viral envelope [23,24], which, together with the rapid mode of action, may explain the inhibition of cell-to-cell spread of HCMV, HSV-1, and HSV-2 in our experiments. It might be sufficient to compromise the integrity of the viral envelope at a given point to prevent further entry, whereas nAbs need to neutralize the majority of entry-related epitopes to prevent infection. This makes targeting the viral envelope a promising strategy for drug development. In summary, the key advantage of CLR01 over nAbs seems to be the interaction with the viral envelope, which may prevent fusion with the cell membrane. Interference with fusion processes by targeting the viral envelope with virucidal molecules has been demonstrated before and appears extremely efficient and applicable against a broad range of enveloped viruses [55].

### 4.3. Outlook

The efficient inactivation of viral particles by CLR01 underscores the remarkable potential of molecular tweezers against herpesviruses and could create new opportunities for studying herpesvirus entry and cell-to-cell spread, in addition to interesting clinical applications. The mechanism by which CLR01 inhibits viral infection may also represent a broad effective strategy against enveloped viruses, from which they can hardly escape.

## Supplementary Materials

The following are available online at https://www.mdpi.com/article/10.3390/v13091685/s1, Figure S1: Pretreatment of cells with CLR01 does not alter infection, Figure S2: CLR01 inhibits direct cell-to-cell spread of HCMV strain TB40/E.
