# Supplementary material for: The Molecular Tweezer CLR01 Inhibits Antibody-Resistant Cell-to-Cell Spread of Human Cytomegalovirus
Source: Viruses. 2021 Aug 25;13(9):1685. doi: 10.3390/v13091685 (PMC8472163; doi:10.3390/v13091685)
Supplement: Supplementary file 1 [file viruses-13-01685-s001.zip › viruses-1343652-supplementary.pdf]

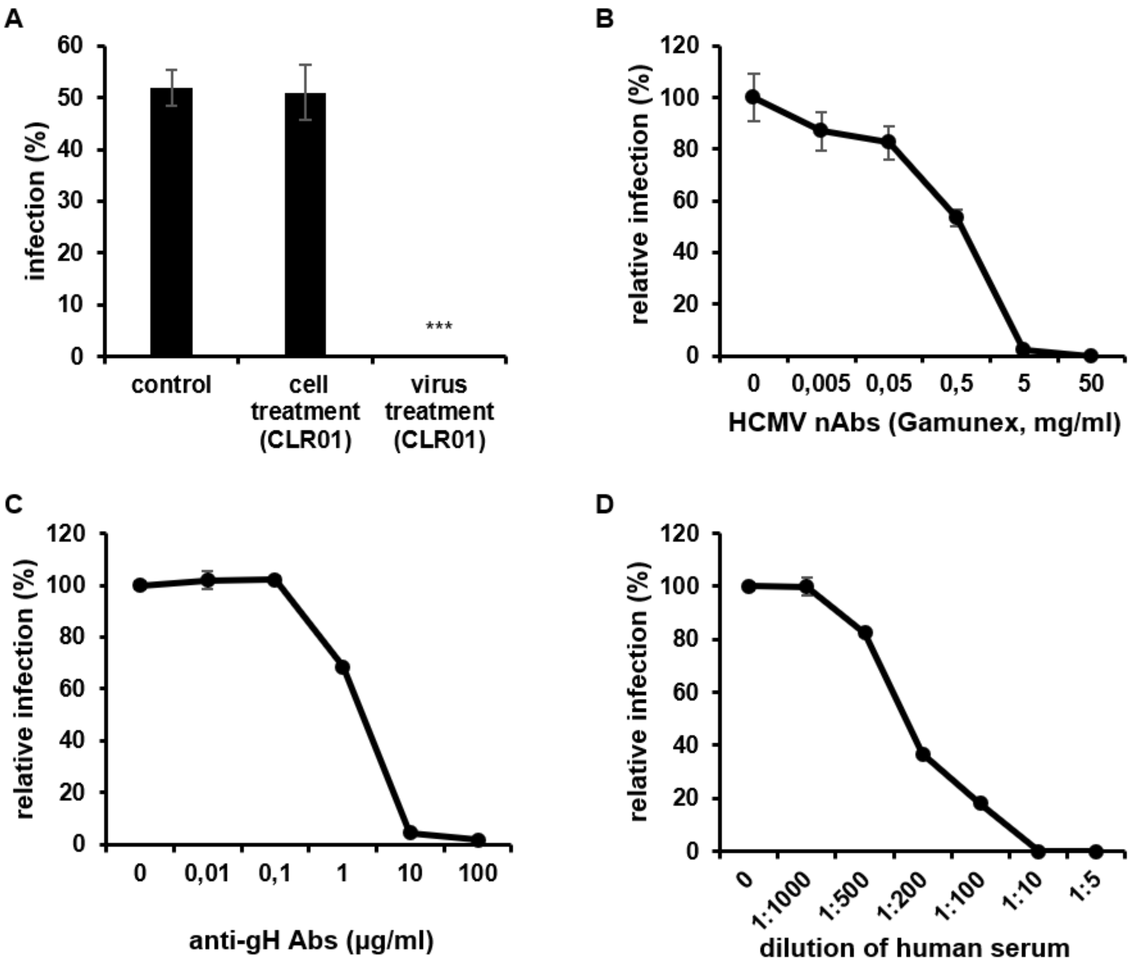

Figure S1: A: Pretreatment of cells with CLR01 does not alter infection. Human fibroblasts and cell-free virus particles of HCMV (strain TB40/E, corresponding to an infection of about 50%) were pretreated with 50 µM CLR01 for 1 h at 37°C. Then, pretreated cells were washed and infected with untreated virus for 1 h at 37°C, whereas pretreated HCMV particles were added to untreated cells and incubated for 1 h at 37°C. Infection was determined by indirect immunofluorescence of HCMV IE antigen 24 hpi. Means ± SDs of infection (%) from triplicate infections. *, $P < 0.01$; **, $P < 0.001$; ***, $P < 0.0001$**. B-D: Titrations of HCMV neutralizing antibodies against cell-free TB40/E.** Cell-free virus of HCMV strain TB40/E (corresponding to an infection of about 50%) was titrated against (B) Gamunex (HCMV nAbs), (C) anti-gH Abs and (D) elite human serum with outstanding HCMV neutralization capacity and incubated for 1 h at 37°C with indicated concentrations before addition to human fibroblasts for 1 h at

37°C. Infection was determined by indirect immunofluorescence of HCMV IE antigen 24 hpi. Means ± SDs of infection (%) from triplicate infections.

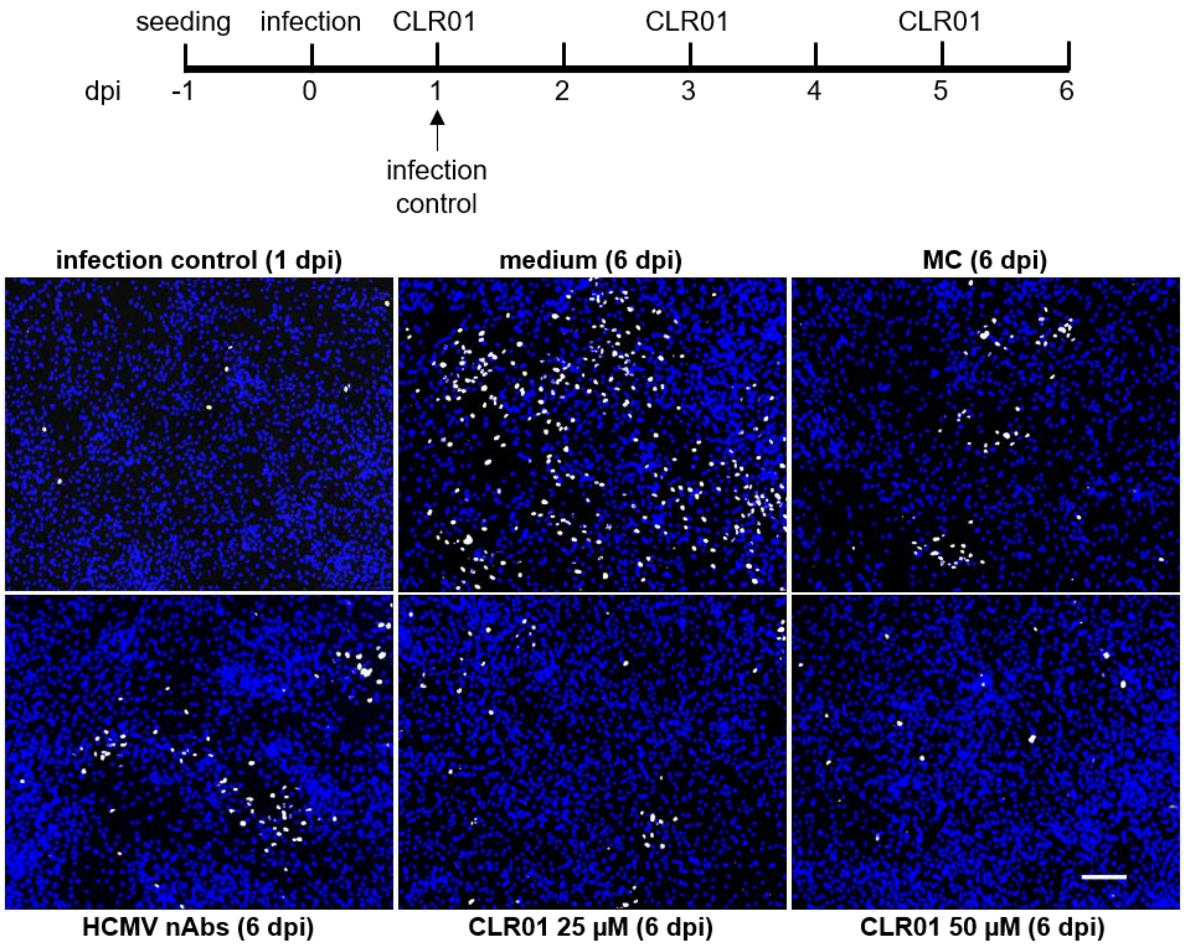

Figure S2: CLR01 inhibits direct cell-to-cell spread of HCMV strain TB40/E. Human fibroblasts were infected with HCMV strain TB40/E (corresponding to an infection of about 0.1 – 1%). Different conditions were applied (medium, methylcellulose [MC] overlay, HCMV neutralizing antibodies [nAbs; 5 mg/ml Gamunex], and indicated concentrations of CLR01) 1 dpi and renewed every 48 h. Focal growth was determined by indirect immunofluorescence of HCMV IE antigen at 6 dpi. Initial infection was controlled by detection of HCMV-positive cells at 1 dpi (infection control). Blue, DAPI-positive cells; white, HCMV-positive cells. Scale bar is 200 µm.
